# Tickling induces a unique type of spontaneous laughter

**DOI:** 10.1098/rsbl.2024.0543

**Published:** 2024-11-20

**Authors:** Roza G. Kamiloğlu, Rui Sun, Patrick Bos, Florian Huber, Jisk Jakob Attema, Disa A. Sauter

**Affiliations:** ^1^ Department of Psychology, University of Amsterdam, Amsterdam, The Netherlands; ^2^ Department of Experimental and Applied Psychology, Vrije Universiteit Amsterdam, Amsterdam, The Netherlands; ^3^ Roman Family Center for Decision Research, Chicago Booth School of Business, Chicago, IL, USA; ^4^ Netherlands eScience Center, Amsterdam, The Netherlands; ^5^ Centre for Digitalization and Digitality, University of Applied Sciences Düsseldorf, Düsseldorf, Germany

**Keywords:** tickling, laughter, acoustics, machine learning, spontaneous

## Abstract

Laughing is ubiquitous in human life, yet what causes it and how it sounds is highly variable. Considering this diversity, we sought to test whether there are fundamentally different kinds of laughter. Here, we sampled spontaneous laughs (*n* = 887) from a wide range of everyday situations (e.g. comedic performances and playful pranks). Machine learning analyses showed that laughs produced during tickling are acoustically distinct from laughs triggered by other kinds of events (verbal jokes, watching something funny or witnessing someone else’s misfortune). In a listening experiment (*n* = 201), participants could accurately identify tickling-induced laughter, validating that such laughter is not only acoustically but also perceptually distinct. A second listening study (*n* = 210) combined with acoustic analyses indicates that tickling-induced laughter involves less vocal control than laughter produced in other contexts. Together, our results reveal a unique acoustic and perceptual profile of laughter induced by tickling, an evolutionarily ancient play behaviour, distinguishing it clearly from laughter caused by other triggers. This study showcases the power of machine learning in uncovering patterns within complex behavioural phenomena, providing a window into the evolutionary significance of ticking-induced laughter.

## 1. Introduction

Laughter has deep evolutionary roots: many mammals, including chimpanzees, squirrel monkeys and dogs produce laughter-like vocalizations during play [1]. In humans, laughing is one of the first complex social behaviours we engage in: human infants start to laugh within weeks of birth [2]. Even congenitally deaf individuals, who have not heard the vocalizations of themselves or others, produce laughter consisting of fundamentally similar acoustic structures to hearing individuals [3,4]. These findings suggest that innate mechanisms play a key role in this vocal behaviour; laughter is clearly embedded in our biology. However, this does not mean that laughter is fixed or that all laughing sounds the same: we might politely snigger when a friend tells a bad joke, burst into a deep belly laugh at a comedic scene in a movie and giggle when tickled by a friend. Are there systematic differences between laughs? Given its prevalence and significance in human life, examining distinctions among laughter types is essential for understanding their unique functions.

Attempts to differentiate between different kinds of laughter have focused on perceptual features, that is, what distinctions listeners can make. Using laughter recorded in tightly controlled laboratory settings, researchers have found that listeners can tell whether laughter occurs between friends or strangers [5] and whether the laughing person is from their own cultural group or not [6]; perceivers can categorize laughter in terms of emotion states like joyful and mocking [7,8]; and distinguish spontaneous (i.e. genuine, involuntary) from volitional (i.e. deliberate, voluntary) laughter [9]. Despite the documentation of these perceptual distinctions in laughter, however, we know little about the production of laughter. To better understand laughter, we need to expose how laughter is acoustically formed as a response to elicitors in its natural environment. By leveraging machine learning techniques, we can uncover patterns within the acoustic forms of laughter based on their production contexts.

This approach builds on the ethological method, which examines behaviour in its natural context and seeks to establish the proximate triggers that elicit a given response [10,11]. It has yielded important insights into the behaviours of non-human animals, including Goodall’s meticulous observations of chimpanzee behaviour [12] and the early imprinting studies of Lorenz that established critical periods in animal development [13]. Most ethological studies, however, are based on small samples and are limited to low-dimensional data obtained with methods like note-taking or direct observation. Given recent technological advancements, computational methods can establish statistical regularities in behaviours in relation to their natural production contexts at a larger scale [14]. In combination with the availability of rich video and audio samples from daily life, this allows large-scale examinations to capture situational features that systematically elicit particular behaviours. In the current study, we apply a computational ethology approach to laughter, thus investigating the classification of laughter acoustics based on production contexts in a bottom-up manner.

### (a) The present study

To test whether there are fundamentally different kinds of laughter, we analysed a diverse dataset of spontaneous laughs (*n* = 887) sampled from a wide range of everyday situations [15]. Using a supervised machine learning model, we first established that laughter possesses a distinct acoustic profile, accurately differentiating it from other non-verbal vocalizations with 93% accuracy (see electronic supplementary material, Text S1). This provided a foundation for further analysis of potential laughter subtypes.

Building on this, we used machine learning techniques to identify potential patterns within the laughter vocalizations. We identified the production context of each laugh and used a machine learning model to examine if acoustic features could distinguish between laughter types based on these contexts. Finally, we conducted two listening experiments to investigate human perception of these acoustic distinctions. The first aimed to determine if listeners could accurately identify a specific laughter type, while the second explored how laughter types relate to judgments of vocal control and other perceptual features.

## 2. Machine learning experiment

**Figure 1.**
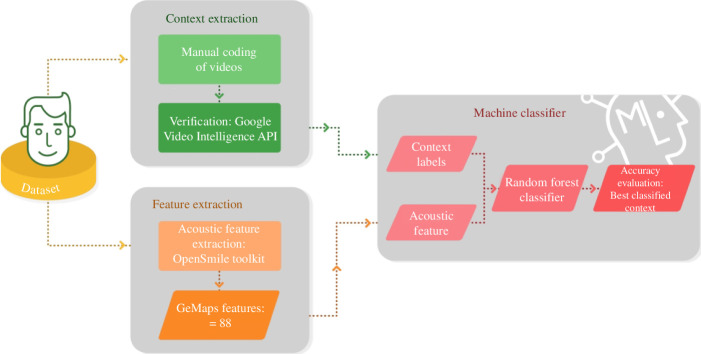
Analysis framework. We (1) identified the production context of laughs; (2) extracted a large number of acoustic features from each audio file; (3) used a random forest classifier to test how well the acoustic structure of laughs could be differentiated based on the situations in which they occurred.

We sought to test whether there is systematic variation between laughs. We first identified the production context in which each laugh occurred from the video clips. We then used a machine learning model—to examine if we could distinguish between the laughter types based on their acoustic features, according to the production context of each laugh. This framework is illustrated in figure 1.

### (a) Methods

#### (i) Laughter samples

We compiled 887 laughs from real-life videos on YouTube (https://www.youtube.com) [15]. Each video contained a single, clearly audible laugh, lasting approximately 10 s with the laughter occurring at the end. Naive research assistants were tasked with searching for videos of laughter, adhering to three strict inclusion criteria: (i) the presence of a clearly audible laugh, (ii) a clear and unambiguous eliciting real-life situation, and (iii) only one person vocalizing. To ensure the spontaneity of the laughter events, we adopted a selection process [16,17]. This approach prioritized videos capturing sudden events, minimizing the chance of posed or managed laughter. Research assistants selected only videos they were highly confident reflected spontaneous laughter, based on situational clarity and their judgement.

#### (ii) Production context of laughs

To examine how laughter varies with context, we analysed the situations in which laughs occurred without any pre-defined categories. Naive research assistants manually coded the videos by observing and noting objectively detectable features of each situation that led to laughter. From these systematic observations, we identified patterns and clustered the laughs based on the primary triggers. To ensure accuracy, these classifications are verified using the Google Video Intelligence API. This revealed four distinct context types (see electronic supplementary material, Text S2 for definitions): being tickled (e.g. a playful sibling ambush or a friend’s unexpected tickle attack, *n* = 241), watching something funny (e.g. a comedy sketch or a humorous scene in a movie, *n* = 276), witnessing someone else’s misfortune (e.g. seeing someone mistakenly pouring salt instead of sugar into their coffee, *n* = 175) and hearing a verbal joke (e.g. a classic ‘knock-knock’ joke or a witty one-liner, *n* = 195). These contexts showed balanced gender distribution (see electronic supplementary material, figure S3 for descriptive information) and were classified with 68% accuracy by the API with low confusion between them, confirming their distinctiveness (see electronic supplementary material, Methods Text S3 for details). Our aim was to capture the primary trigger of the laughter in each clip, allowing us to investigate whether laughter produced in these four kinds of situations would be acoustically discriminable.

#### (iii) Classification experiment

We used machine classifiers to test whether the acoustic forms of laughter would differ between behavioural contexts using a RF algorithm [18]. This machine learning approach is particularly suitable for this dataset, which comprises a relatively large number of features (88) compared with the number of laughter instances (887), and allows us to identify patterns in the acoustic features associated with different laughter contexts. We extracted 88 acoustic features from each of the laughter files (*n* = 887) using the GeMaps library [19] of the OpenSmile toolkit [20]. This feature set encompasses a wide range of acoustic parameters relevant to vocalization analysis. These features were used as input in an RF classifier for testing whether laughs produced in the different contexts would be acoustically distinguishable. In applying the machine classifier, we split the data as 30% test and 70% training data, set 1000 trees in the forest with maximum of 60 features for splitting a node and maximum of 55 levels in each decision tree. To better understand the underlying acoustic signatures of laughter elicited in different situations, we also identified which acoustic features are most effective in distinguishing them by examining feature importance using a SHAP (SHapley Additive exPlanations) analysis [21].

### (b) Results

#### (i) Tickling-induced laughs are distinct from other types of laughter

An RF classifier demonstrated that overall accuracy (40%) was above the chance level (approx. 25%). However, laughter produced when being tickled was considerably better differentiated than laughter from the other contexts, in that the model produced fewer false classifications for laughs elicited by someone being tickled. We implemented a grid search methodology for further performance tests. This approach allowed us to optimize the model through an extensive evaluation of different parameter combinations. The overall accuracy slightly increased (48%), and tickling laughs were again the best-classified type of laughter, suggesting that the model was robust and that laughter elicited by tickling is clearly distinct from laughter elicited by other triggers. Evaluation metrics for the default classification model are provided in table 1. To ensure the generalizability of our findings and account for dataset variability, we performed cross-validation analyses, which confirmed the model’s robustness and the reliability of acoustic distinctions between laughter types (see electronic supplementary material, Text S4).

**Table 1.** Evaluation metrics (precision, recall, F1-score) for acoustic classification of laughs in four contexts. Scores range from 0 to 1, with higher values indicating better model performance.

	precision	recall	F1-score
being tickled	0.63	0.58	0.60
funny stimuli	0.38	0.64	0.48
misfortune of others	0.30	0.16	0.21
verbal joke	0.14	0.08	0.10

**Figure 2.**
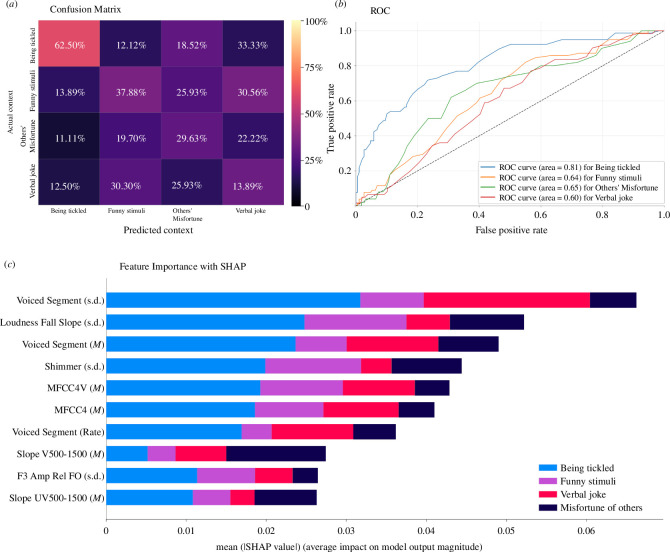
Acoustic classification of laughter elicited in four contexts. (*a*) Confusion matrix of the default classifier showing correct and incorrect prediction (in percentages); (*b*) ROC curves, which depict the true positive rate against the false positive rate of the classifier, illustrates its accuracy and performance; (*c*) SHAP Value Analysis for Laughter Type Classification, illustrating the mean SHAP values for the 10 most impactful acoustic features across four laughter contexts.

The most differentiating acoustic features included the length of voiced segments (mean, s.d. and rate), mean of mel-frequency cepstral coefficient 4 (MFCC4; i.e. short-term spectral based features), amplitude variation of the vocal cord vibration (i.e. s.d. of shimmer) and variability of loudness. For example, being tickled resulted in laughs with fewer rhythmic bursts (akin to voice segment rates) with an average of 2.68 bursts (s.d. = 1.38), as compared to laughs elicited by funny stimuli (*M* = 3.79, s.d. = 1.95), verbal jokes (*M* = 4.30, s.d. = 1.94), or witnessing others’ misfortune (*M* = 3.92, s.d. = 1.63). Figure 2 presents the confusion matrices for the classifications, the ROC curve (receiver operating characteristic; a graphical plot that illustrates the diagnostic ability of a binary classifier system as its discrimination threshold varies), and the rankings of the 10 most important acoustic features for classification. Electronic supplementary material, table S7 provides means and s.d. values of the acoustic features for laughs produced in the four behavioural contexts.

## 3. Listening experiment 1

Do the distinct acoustic features of laughter produced when being tickled also allow listeners to accurately infer what kind of event triggered a given laugh? We tested whether listeners would be able to accurately identify whether a laugh was produced in the context of tickling from just hearing it (preregistration at https://osf.io/tke54/).

### (a) Methods

#### (i) Participants

We recruited listeners (*n* = 201; 117 women, 74 men, seven non-binary, three preferred not to report their gender; *M*_age_ = 20.51 years, s.d._age_ = 3.60). Participants who reported having no hearing impairment were recruited via the subject pool at the University of Amsterdam, as well as our personal networks and social media.

### (b) Materials and procedure

#### (i) Laughter clips

We used the clips collected for machine learning analysis. In total, 241 laughter clips were produced in tickling and 646 laughter clips were produced in non-tickling contexts.

#### (ii) Experimental procedure

Each participant played 30 laughs from the corpus. For each participant, half of the laughter clips they heard were produced while being tickled; the other half consisted of five laughter clips from each of the non-tickling categories (i.e. funny stimuli, others’ misfortune, verbal jokes). The laughs were randomly selected from the full corpus of clips to ensure that each clip was presented to at least one listener. After listening to each clip, participants were asked to judge whether they thought that the laugh was produced while the laughing person was being tickled or not by selecting ‘Yes’ or ‘No’. The presentation order of the clips was randomized for each listener. There were no time constraints for completing the experiment, and participants were allowed to listen to each clip as many times as they wanted.

#### (iii) Statistical analysis

The sensitivity index d-prime was used to quantify listeners’ ability to judge whether each laugh was produced in a tickling context. It controls for individual biases for using one of the response options and is calculated as *Z*-transformed hit rates minus *Z*-transformed false alarm rates [22]. Hit and false alarm rates with extreme values (i.e. 0 or 1) return an error when *Z*-transformed. Those cases were, as is commonly done, adjusted by replacing rates of zero with 0.5/*n* and rates of 1 with (*n*−0.5)/*n* where *n* is the number of signal/noise trials [23]. D-prime scores were calculated for each listener to test whether human listeners are able to detect at better-than-chance levels whether laughter was produced while the laughing person was being tickled or not. We compared d-prime scores to chance (denoted with a d-prime of zero) using Wilcoxon signed-rank test, because the d-prime scores were not normally distributed, as shown by a Kolgomorov–Smirnov test (*D*(200) = 0.067, *p* = 0.29). To further examine trial-level performance, we conducted a multilevel logistic regression with laughter context (tickling versus other) as a fixed effect and participant and audio clip as random effects.

### (c) Results

**Figure 3.**
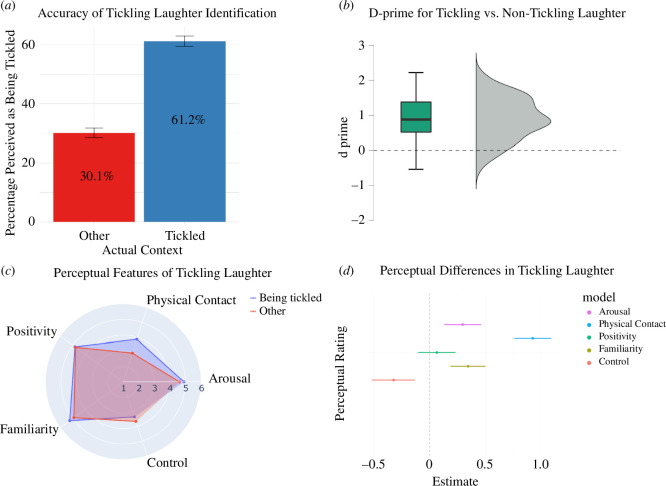
(*a*) Bar plot displaying the percentage of times laughter was correctly or incorrectly perceived as tickling-induced, based on its actual context (tickling versus other). (*b*) Rain cloud plot of d-prime scores indicating perceptual judgements of whether laughs were produced while the person was being tickled or not. For the box plot, the vertical line is the median, box edges indicate the 25–75% range and the whiskers indicate minimum and maximum. The dashed line shows the chance level. (*c*) Radar plot showing the means of five perceptual judgements rated from 1 (lowest) to 7 (highest). (*d*) Effect size estimates from the LMM analysis, highlighting significant perceptual differences between tickling-induced and other types of laughter in terms of five perceptual features.

Figure 3*a* illustrates that participants correctly identified tickling laughter 61.2% of the time (95% CI [59.5–63%]) and misidentified non-tickling laughter as tickling 30.1% of the time (95% CI [28.5–31.7%]). Wilcoxon signed-rank test showed that listeners were able to correctly judge whether laughter was produced during tickling, with performance significantly better than random guessing (*V* = 18499, Mdn = 0.88, *p* < 0.001; see figure 3*b*). Multilevel logistic regression showed a significant effect of laughter context on perception (*β* = 1.36, s.e. = 0.11, *z* = 11.95, *p* < 0.001), with the odds of perceiving laughter as tickling being 3.88 times higher for actual tickling laughter (95% CI [3.11–4.85]). The model also accounted for variability across participants and clips (s.d.s = 0.33, 0.27, respectively). Both d-prime and regression analyses confirm listeners’ ability to differentiate whether someone is being tickled or not, just from hearing them laugh.

## 4. Listening experiment 2

We wanted to explore if the reason behind a laugh—such as being tickled or not—could influence how people perceive the laugh. In a second preregistered perception experiment (preregistration at https://osf.io/r48eg), a new set of naive participants listened to laughs produced in the four contexts and were asked to evaluate certain perceptual features.

### (a) Methods

#### (i) Participants

We recruited a set of listeners (*n* = 210; 110 women, 90 men, five non-binary, one other, four preferred not to report their gender; *M*_age_ = 20.54, s.d._age_ = 3.14) who report having no hearing impairment via the subject pool at the University of Amsterdam and through our personal networks and social media.

### (b) Materials and procedure

#### (i) Laughter clips

Full laughter dataset including being tickled (*n* = 241) and non-tickling (*n* = 646) laughs was used.

#### (ii) Experimental procedure

Participants were asked to listen to 30 clips (15 tickling and 15 non-tickling laughter) randomly selected from the larger pool, and evaluate, on a 1–7 Likert scale, to what extent they thought the laughing person was aroused, positive and in control of their voice. Listeners also rated how likely they thought it was that each laugh was produced in a context involving people familiar to each other, and how much physical contact they thought there was (if any) between the people in the situation that elicited the laughter (see electronic supplementary material, Text S5 for details).

#### (iii) Statistical analysis

The following linear mixed model (LMM) was used to test whether the production context of laughs (being tickled versus not) would predict the perceptual ratings for each of the five measures: positivity, arousal, control, physical contact and familiarity.


PerceptualRating∼Context+(1|ListenerID)+(1|ClipID),data=listeningdata

The model allowed us to define fixed and random effects, and assign random intercepts for Clip ID and Listener ID. We used R and lme4 [24] to perform a linear mixed effects analysis. We also used the lmerTest package [25] to estimate denominator degrees of freedom using Satterthwaite’s approximation and to generate *p*-values for mixed models. We conducted a complementary cumulative logit model analysis, acknowledging the ordinal nature of the Likert scale data.

### (c) Results

Figure 3*c* provides a radar plot of the mean ratings for each perceptual measure for being tickled versus non-tickling situations; the means for these perceptual measures are available in electronic supplementary material, figure S9. The LMM results showed that laughs produced in tickling contexts were perceived as higher in arousal (*β* = 0.30, 95% CI [0.13, 0.46], *p* < 0.001) and less controlled (*β* = − 0.32, 95% CI [−0.52, −0.13], *p* < 0.001) than other laughs (see figure 3*d*). Moreover, perceivers thought tickling laughs were likely to be produced in situations involving more physical contact (*β* = 0.93, 95% CI [0.76, 1.09], *p* < 0.001), and people with higher familiarity (*β* = 0.34, 95% CI [0.19, 0.50], *p* < 0.001). While listeners’ perceptions may be influenced by associations with tickling, the consistent acoustic distinction across analyses suggests our classification is primarily driven by acoustic features. Perceived positivity did not differ between laughs produced in the context of being tickled and other situations (*β* = 0.07, 95% CI [−0.10, 0.24], *p* = 0.44). The complementary cumulative logit model confirmed that tickling-induced laughter as more arousing, less controlled and linked to more physical contact and familiarity, with no difference in perceived positivity (see electronic supplementary material, Text S6).

## 5. Discussion

Are there different kinds of laughter? Our findings demonstrate that laughter resulting from tickling is acoustically distinct from laughter triggered by verbal jokes, witnessing someone else’s misfortune or watching something funny. Furthermore, naive listeners can accurately discern tickling-induced laughter without access to any visual cues. Tickling is a form of play behaviour that has been observed across various animal species, including Barbary macaques and chimpanzees and serves as the closest link between human laughter and other mammalian play vocalizations [1,26,27]. In these species, tickling often elicits vocalizations akin to laughter, which share certain acoustic features [27]. These shared features suggest a phylogenetically conserved behaviour that dates back to the last common ancestor of great apes, approximately 10 million years ago [28]. In contrast, laughter resulting from more complex stimuli, such as verbal irony, parody or jokes, involves a level of cognitive processing that is likely unique to humans [29]. The present study provides evidence for a fundamental distinction between tickling-induced laughter, an evolutionarily ancient form of vocalization and laughter elicited by more cognitively demanding situations, which may be unique to humans.

The present study points to specific acoustic features that contribute to the distinctiveness of tickling-induced laughter. One of the most salient features was the rate of voiced segments, which refers to the frequency of measurable, periodic vocal fold vibrations (both syllabic and non-syllabic) within each second of a laughter episode. This metric can provide insights into vocal control, although the relationship between voiced segment rate and control may be influenced by various factors, including the presence of non-syllabic vocalizations. Previous research has suggested that spontaneous laughter can exhibit high syllable rates and variable vocal patterns [30,31]. Our metric differs in that it also captures non-syllabic sounds and irregular vocal fold vibrations. We found that tickling-induced laughter displayed higher mean and standard deviation of voiced segment rates compared to other types of laughter, as seen in the classification results (figure 2*c*). This suggests that the variability in vocal patterns may contribute to the distinctiveness of tickling-induced laughter. This aligns well with the results from the listener experiments, which showed that tickling-induced laughter is perceived as less controlled. Moreover, tickling-induced laughter was also perceived as more aroused than other laughs, but no differences were found in perceived positivity from other types of laughter. The heightened arousal observed in tickling-induced laughter, with its reduced vocal control, suggests an automatic response beyond its valence directly tied to the act of being tickled.

Finally, the present approach demonstrates how applying the tools of machine learning to rich bodies of video and audio data can reveal systematicity in socially complex and high-dimensional behavioural domains like human laughter. Technological advancements in recent years allow us to study behaviours in the contexts in which they naturally occur, with machine learning illuminating differentiations that were previously unknown. By investigating systematic associations between the acoustic structure of laughs and the situations in which they were produced, this approach reveals the uniqueness of tickling-induced laughter.

## Data Availability

All data and reproducible scripts are publicly available at [32]. Supplementary material is available online [33].
